# Is it possible to substitute the monofilament test for the Ipswich Touch Test in screening for peripheral diabetic neuropathy?

**DOI:** 10.1186/s13098-020-00534-2

**Published:** 2020-03-31

**Authors:** Luz Marina Alfonso Dutra, Mirian Conceição Moura, Flaviene Alves do Prado, Giselle De Oliveira Lima, Manuela Costa Melo, Rubens Nelson Morato Fernandez, Maria Rita Carvalho Garbi Novaes

**Affiliations:** 1 Department of Health, Brasília, Federal District Brazil; 2 Center of Neuromuscular Diseases, Hospital de Apoio de Brasilia, Brasília, Federal District Brazil; 3 Higher School of Health Sciences, Brasilia, Federal District Brazil; 4 University of Brasília, Brasília, Federal District Brazil

**Keywords:** Adult health, Diabetic foot, Diabetes mellitus, Diabetes neuropathies, Secondary care

## Abstract

**Background:**

This study aimed to assess the agreement and efficacy of the Ipswich Touch Test compared to the monofilament test in individuals with type 2 diabetes.

**Materials and methods:**

A cross-sectional and analytical study was conducted. The inclusion criteria were patients with type II diabetes (n = 250) who did not present ulcers or amputation in either foot. The exclusion criteria were as follows: patients who presented sequelae of cerebrovascular disease or other neurological pathologies, as well as diagnoses of malignancy, alcohol abuse, liver cirrhosis, hepatitis B, AIDS, hypothyroidism, chronic kidney disease or lupus erythaematosus, as these clinical conditions could influence or bias the results (Won and Park in Endocrinol Metab 31:230–238, 2016). Sensitivity, specificity, predictive values, likelihood ratios, and Kappa index were calculated. Other factors assessed were glycated haemoglobin and body mass index.

**Results:**

Most of the participants were female (71.2%), and glycated haemoglobin (HbA1c) was greater than 7% in 54.4% of the patients. The mean age was 59.43 years, and the mean time since diagnosis was 12.38 years. The Kappa index was 0.819 (p < 0.001), and the Ipswich Touch Test had a sensitivity of 83.33%, a specificity of 97.66%, a positive predictive value of 85.71%, a negative predictive value of 97.21%, a positive likelihood ratio of 30.19%, and a negative likelihood ratio of 0.17%. The level of significance was 5% in this study.

**Conclusion:**

The Ipswich Touch Test resented good agreement and efficacy compared to the gold standard—the 10 g monofilament test.

## Background

Diabetic neuropathy is a complication of chronic diabetes and results from heterogeneous conditions that impair nerve conduction [1, 2]. Distal symmetric polyneuropathy (DSP) is the most common complication of both types of diabetes [3] and accounts for peripheral nerve dysfunction in diabetic patients after the exclusion of other types of diabetes, such as traumatic or neoplastic conditions, and other systemic diseases [4]. Distal symmetric polyneuropathy (DSP) depends on the length of the neuron [3], and may account for 75% of diabetic neuropathies [5, 6]. DSP is an important cause of foot ulceration and Charcot arthropathy [7], leading to amputation and increases in economic costs [8].

Manifestations of DSP can vary from subclinical symptoms to painful ones, such as burning, tingling, itching or prickling [3]. The pain is insidious and increases in severity due to the impairment of the peripheral neurons. It can affect the quality of life and mobility of patients and can lead to mood disorders and relationship problems in patients [8, 9].

The guidelines of the American Diabetes Association (ADA) and the Brazilian Diabetes Society (SBD) recommend that all people with diabetes should receive at least one foot assessment annually to identify risk factors for ulceration or amputation and that these foot assessments should begin immediately after diagnosis with type 2 diabetes and 5 years after diagnosis with type 1 diabetes [10, 11].

The Ipswich Touch Test (IpTT) [12] is a simple way to conduct a screening test, principally in places with poor resources. Furthermore, the IpTT can be carried out by any trained health professional and simply involves lightly touching the tips of the first, third and fifth toes and the dorsum of the hallux with the index finger for 1–2 s. Further studies in different locations are necessary to gather more information about the IpTT and to validate this test [13].

Accordingly, this study aimed to evaluate the agreement and efficacy of the IpTT in relation to the monofilament test in individuals with type 2 diabetes, to contribute to the existing literature by providing evidence for a simpler screening technique, and to provide a test that can be undertaken at an early stage to avoid ulceration.

## Methods

### Participants

In total, 250 individuals who attended a diabetes outpatient centre were assessed. The inclusion criteria were as follows: individuals with type 2 diabetes who did not present ulcers or amputation in either foot. The exclusion criteria were as follows: patients who presented sequelae of cerebrovascular disease or other neurological pathologies, as well as diagnoses of malignancy, alcohol abuse, liver cirrhosis, hepatitis B, AIDS, hypothyroidism, chronic kidney disease or lupus erythaematosus, as these clinical conditions could influence or bias the results. [14].

### Study design and setting

This is a cross-sectional and analytical study conducted at a centre specializing in diabetic patients located in the centre of Brasília. The centre also specializes in patients with foot pathologies. The data were collected between September 2017 and March 2018.

### Data collection

The individuals were assessed in a quiet environment to avoid any noise that could have interfered with the results. The tests were conducted by physicians and nurses; a total of 6 professionals were trained to carry out the tests.

The gold standard test for the triage of the risk of ulceration is the 10 g monofilament test. It must be applied perpendicularly for a period of approximately 2 s in the base of the hallux in the 1st, 3rd, and 5th metatarsi heads. The patient must be asked to say “yes” when the area being tested is touched with a strength that is just enough to provoke the monofilament to curve for 2 s. A simulation of the application and another concrete application in the same areas confirm that the patient has identified the site tested only when two answers are correct; any insensitive area indicates loss of plantar protective sensitivity. The test is not recommended on sites where there are scars, callosities, hyperkeratosis, since these may lead to errors in the results.

The monofilament test was complemented with a 125 Hz tuning fork placed on the dorsum of the hallux for a period of 2 s as a means of complementing the test [11, 15]. The monofilament fibre was used to assess a maximum of 10 patients/day.

The IpTT was undertaken using the tip of the index finger for a period of approximately 2 s on the tips of the first, third and fifth toes.

When both tests presented 2 negative points, the results were considered to be negative. Individuals were asked to close their eyes when they received the tests and to respond with the word “yes” when they felt the touch. Tests were carried out by experienced professionals who had been trained and worked in the institution for a long time, in secondary health care. Each participant underwent the monofilament test at least twice, and whenever the test was inconclusive or its results unclear, another professional was asked to carry out an evaluation, to avoid bias in the results.

The values for glycated haemoglobin and Body mass index (BMI), taken from electronic medical records, were also taken into account.

To classify the severity of the symptoms, the researchers used the instrument for screening symptoms developed by the Federal District’s State Department of Health and in conjunction with the Brazilian Diabetes Society, following the recommendations of the International Working Group on the Diabetic Foot (IWGDF) [16, 17]. The clinical symptoms were assessed in relation to pain, burning and tingling and were compared using a visual analogue scale from 0 to 10, with 10 being the maximum pain reported.

These algorithms allow the results to be classified as follows: symptoms scoring from 0 to 2 points are normal; from 3 to 4 points are mild; from 5 to 6 points are moderate; and from 7 to 9 points are severe.

### Data analysis

The Kappa index was used to describe the agreement between the monofilament test and IpTT. To measure the accuracy of the tests, the researchers analysed sensitivity and specificity, likelihood ratios and predictive values; the Chi squared test was used to compare the tests with the neuropathic symptoms. The level of significance used throughout the study was 5%.

## Results

In the sample study, most individuals were female, obese, not using insulin and not meeting the goals for glycaemic control, as indicated by an HbA1c value higher than 7. The mean age was 59.43 years, and the mean time since diagnosis was 12.38 years—standard deviation (SD)—10.52, as shown in Tables 1 and 2.

**Table 1:** Descriptive analysis of the qualitative variables of individuals with diabetes (n = 250)

Variable	N	Percentage
Sex		
Male	72	28.8
Female	178	71.2
Insulin		
Yes	118	47.2
No	132	52.8
HbA1c		
Normal < 7	102	40.8
Decompensated > 7	136	54.4
Absent	12	4.8
BMI		
Normal	34	13.6
Pre-obesity	61	24.4
Obesity I	66	26.4
Obesity II	41	16.4
Obesity III	23	9.2
Absent 25		10
Age range^a^		
Below 60	112	44.8
Above 60	138	55.2

*HbA1c* glycated hemoglobina (%), *BMI* body mass index

^a^Years

**Table 2:** Descriptive analysis of the quantitative variables of individuals with diabetes type 2 (n = 250)

Variable	Mean	Standard deviation	Minimum	Maximum
Age^a^	59.43	10.78	25.0	86.0
Time since diagnosis^a^	12.38	8.88	0.4	66.0
Glycated hemoglobin^b^	7.83	1.65	5.0	15.0
BMI	32.10	8.10	19.1	64.7

*BMI* body mass index

^a^Years

^b^% percentage

Using the monofilament test as the gold standard, it was possible to calculate various indicators for the IpTT (sensitivity, specificity, and predictive values).

Out of a total of 250 individuals with diabetes, 36 individuals had a loss of plantar protective sensitivity estimated by the gold standard test (monofilament), which is a prevalence of 14.40% for the loss of sensitivity. The IpTT presented good results, with a sensitivity of 83.33% and specificity of 97.66%. Thus, if the patient had loss of plantar protective sensitivity, the IpTT presents an 83.33% probability of identifying the loss of sensitivity (i.e., that the result is truly positive—TP), and if the patient does not have this loss, the IpTT has a 97.66% probability of identifying this absence (i.e., that the result is truly negative—TN). The positive and negative predictive values were 85.71% and 97.21%, respectively. Therefore, among the individuals with positive results in the IpTT, the chance of the individual genuinely presenting loss of plantar protective sensitivity is 85.71%, and among the individuals with negative results, the chance of the individual genuinely presenting a loss is 97.21%.

The IpTT was highly accurate when compared with the monofilament test (positive likelihood ratio = 35.61), indicating that the chance of a patient having a loss of sensitivity if the result of the IpTT was positive is 35.61 times greater than a patient with a negative result for the IpTT (Table 4). Table 3 presents a 4 × 4 table with the absolute values for patients with and without LOPS.

**Table 3:** Percentage of patients with loss of protective sensation

LOPS with IpTT				
		No	Yes	Total
LOPS with monofilament				
No		209	5	214
Yes		6	30	36
Total		215	35	250

It may be observed that with the prevalence estimated for this study (14.40%), the positive predictive value (PPV) is 85.71%, and the negative predictive value (NPV) is 97.21%, as also shown in Table 4.

**Table 4:** Sensitivity, specificity and predictive values of the IpTT for loss of protective sensation, using the monofilament test as the gold standard, among individuals with diabetes (n = 250)

	IpTT
Sensitivity (%)	83.33
Specificity (%)	97.66
Positive predictive value (%)	85.71
Negative predictive value (%)	97.21
Positive likelihood ratio	30.19
Negative likelihood ratio	0.17

The researchers used the Kappa index to assess the agreement between the two tests, with a result of 0.819 (*p* < 0.001), indicating a high level of agreement between both tests, which was statistically significant at the level of significance of 5% (Table 4).

Using Pearson’s Chi squared test, the researchers classified the symptoms as mild, moderate or severe and related them to loss of protective sensation in each test. As a result, the researchers identified a relationship of significance between loss of plantar protective sensitivity and severe symptoms (p < 0.001).

## Discussion

Previous studies [12, 18] were extremely relevant as support for this work. Their methodology was rigorous, and they were carried out in different environments with different professionals, presenting significant results with regards to their sensitivity and specificity in the use of the monofilament, in addition to their reliability, as indicated by the Kappa levels, in both tests. This investigation showed results similar to those of said studies, with a sensitivity of (83.33%) and a specificity of (97.66%), while the values found by Rayman 2011 were, respectively, 76% and 90%, and those found by Sharma et al. 81.2% and 96.4%.

Therefore, applying the IpTT in this diabetic population, whose sociocultural features are different and whose access to health services is restricted, reiterates the applicability of the test in places that are difficult access or lack proper instruments to screen for ulceration risks.

The study demonstrated that the IpTT is reliable in screening for neuropathy at six points, as the Kappa index was 0.819 (p < 0.001) compared to the 10 g monofilament test, showing excellent agreement. It is important to emphasize that although the monofilament test is an easy instrument to handle and is of low cost, it presents high sensitivity and specificity [19, 20] for diagnosing the presence of peripheral neuropathy. In this study, most of the patients were female, were obese, were not meeting the goals for glycaemic control, were not using insulin, had a mean age of 59.43 years and had a mean time since diagnosis with diabetes of 12 years. The characteristics found in the sample were similar to those of another multicentre study undertaken in Brazil that evaluated the risk factors for ulceration with regard to sex, mean age, time since diagnosis with diabetes and Hba1C values [21]. The mean prevalence of peripheral diabetic neuropathy worldwide varies between 16 and 66% [15, 19]. The present study found a percentage of 14.40% (36) of patients with loss of plantar protective sensitivity (LOPS) with the monofilament test. The lower prevalence found in the present study may be related to the sample size or the need for greater accuracy tests for diagnostic confirmation.

The recommendation both in Brazil and internationally is that patients with type II diabetes should be screened for peripheral neuropathy as soon as they are diagnosed with diabetes [11]; nevertheless, in Brazil, this assessment is rarely carried out by health professionals in primary care due to the lack of instruments such as the monofilament. This negligence to screen for peripheral neuropathy can occur not only because of the lack of instruments but also because of the lack of training of health professionals, who do not know the relevance of this screening. The simple screening method (the IpTT), which presents a good level of agreement and specificity, may serve as a tracking strategy to alert health professionals in places where access is difficult. As patients with diabetes frequently present painful sensory neuropathy and clinical symptoms, such as burning, pain, tingling, and paraesthesia, whose nature is progressive [2, 4, 5, 11, 15], and assessed the neuropathic symptoms and classified them as mild, moderate or severe and related them to loss of sensitivity. We concluded that in both the monofilament test and the IpTT, patients who presented more severe neuropathic symptoms presented a significantly greater loss of protective sensation than patients with lower scores (Table 5).

**Table 5:** Measures of agreement (Kappa index) between the IpTT and monofilament test, and association of these tests with scores for neuropathic symptoms used in assessing loss of plantar sensitivity in diabetic patients—(n = 250)

		Value	*p*
Measures of agreement	Kappa	0.819	< 0.001
Measures of association (monofilament v. neuropathic symptoms)		25.753	< 0.001
(Pearson’s Chi squared test) (IpTT v. neuropathic symptoms)		19.887	< 0.001

A multicentre study showed similar results with 50% of patients presented moderate or severe pain [21]. Another study reported that symptoms are not a reliable indicator of neuronal harm, as some patients with symptoms of severe pain have little sensory deficit, while others—without painful symptoms—have feet that are completely numb [22]. Currently, a confirmed peripheral neuropathy diagnosis is recommended when the patient has alterations in their nerve conduction speed and one or more abnormal symptoms and signals (pain, burning sensation, tingling, others) [23]. However, if the nerve conduction speed of the patient is normal but there are still questions raised by the presence of said signals and symptoms, validated tests with level. A evidences must be used to evaluate small fiber diabetic neuropathy [24].

Considering that peripheral neuropathy can cause ulceration, is a major public health problem due to its negative impact on psychological and physical health, brings greater mortality, and imposes high costs on the state and family [25, 26], it is necessary to encourage health professionals to screen for neuropathy as soon as diabetes is diagnosed in every health service that works with diabetic patients.

### Limitations

This study presents limitations related to its a cross-sectional design. Another limitation is that the tests were undertaken in a single diabetes centre located in Brasília; although it is the only secondary centre in this city, these patients come from various regions or cities located around Brasília, thus characterizing different populations. A further limitation is that the records in the electronic medical records were incomplete regarding BMI and values for HbA1c, vitamin B12 screening was not performed. Few studies have been found, which limits the comparison of our findings with other results.

## Conclusion

We concluded that the results of the IpTT for screening for peripheral neuropathy presented excellent agreement according to the Kappa index—0.819 (p < 0.001)—in relation to the gold standard and that its results are efficient according to the values presented for sensitivity and specificity. As a result, this means of assessment may be recommended in poor areas where the monofilament is not available for screening, as the IpTT is a simple method of identifying the risk of ulceration. We emphasize that, when the result is inconclusive or negative, it is necessary to perform the test with monofilament or to refer to the performance of other tests with greater accuracy.

## Data Availability

The datasets are available upon request from the corresponding author.

## Abbreviations

ADA: American Diabetes Association
BMI: Body mass index
DSP: Distal symmetric polyneuropathy
HbA1c: Glycated haemoglobin
IpTT: Ipswich Touch Test
LOPS: Loss of plantar sensitivity
IWGDF: International Working Group on the Diabetic Foot
PPV: Positive predictive value
PPN: Negative predictive value
SBD: Brazilian Diabetes Society

## References

[1] Apelqvist J (2012). Diagnostics and treatment of the diabetic foot. Endocrine.

[2] Sinnreich M, Taylor BV, Dyck PJ (2005). Diabetic neuropathies: classification, clinical features, and pathophysiological basis. Neurologist.

[3] Tesfaye S, Boulton AJ, Dyck PJ, Freeman R, Horowitz M, Kempler P (2010). Diabetic neuropathies: update on definitions, diagnostic criteria, estimation of severity, and treatments. Diabetes Care.

[4] Bansal V, Kalita J, Misra UK (2006). Diabetic neuropathy. Postgrad Med J.

[5] Albers JW, Pop-Busui R (2014). Diabetic neuropathy: mechanisms, emerging treatments, and subtypes. Curr Neurol Neurosci Rep..

[6] Dyck PJ, Albers JW, Andersen H, Arezzo JC, Biessels GJ, Bril V (2011). Diabetic polyneuropathies: update on research definition, diagnostic criteria and estimation of severity. Diabetes Metab Res Rev..

[7] Smith AG, Singleton JR (2012). Diabetic neuropathy. Continuum (Minneap Minn).

[8] Pop-Busui R, Boulton AJM, Feldman EL, Bril V, Freeman R, Malik RA (2017). Diabetic neuropathy: a position statement by the American Diabetes Association. Diabetes Care.

[9] Sadosky A, Schaefer C, Mann R, Bergstrom F, Baik R, Parsons B (2013). Burden of illness associated with painful diabetic peripheral neuropathy among adults seeking treatment in the US: results from a retrospective chart review and cross-sectional survey. Diabetes Metab Syndr Obes..

[10] American Diabetes Association (2016). Microvascular complications and foot care. Diabetes Care.

[11] Oliveira JEP, Vencio S, editors. Diretrizes da Sociedade Brasileira de Diabetes: 2015–2016. São Paulo: AC Farmacêutica 2016. http://www.epi.uff.br/wp-content/uploads/2013/10/DIRETRIZES-SBD-2015-2016.pdf.

[12] Rayman G, Vas PR, Baker N, Taylor CG, Gooday C, Alder AI (2011). The Ipswich Touch Test: a simple and novel method to identify inpatients with diabetes at risk of foot ulceration. Diabetes Care.

[13] Madanat A, Sheshah E, Badawy EB, Abbas A, Al-Bakheet A (2015). Utilizing the Ipswich Touch Test to simplify screening methods for identifying the risk of foot ulceration among diabetics: the Saudi experience. Prim Care Diabetes.

[14] Won JC, Park TS (2016). Recent advances in diagnostic strategies for diabetic peripheral neuropathy. Kor Endoc Soc Endocrinol Metab.

[15] International Diabetes Federation. Diabetic foot screening pocket chart. Brussels: IDF 2017. https://www.idf.org/our-activities/advocacy-awareness/resources-and-tools/124:diabetic-foot-screening-pocket-chart.html.

[16] Grupo de Trabajo Internacional sobre el Pie Diabético. Guía práctica y específica para el tratamiento y la prevención del pie diabético. Brussels: IDF; 2015. http://iwgdf.org/map-es/.

[17] Calsolari MR, Nogueira-Machado JA, Vilar L, Pedrosa HC, Pedrosa HC, Vilar L, Boulton AJ (2013). Mecanismos fisiopatológicos envolvidos na neuropatia diabética. Neuropatias e pé diabético.

[18] Sharma S, Kerry C, Atkins H, Rayman G (2014). The Ipswich Touch Test: a simple and novel method to screen patients with diabetes at home for increased risk of foot ulceration. Diabet Med.

[19] Pham H, Armstrong DG, Harvey C, Harkless LB, Giurini JM, Veves A (2000). Screening techniques to identify people at high risk for diabetic foot ulceration: a prospective multicenter trial. Diabetes Care.

[20] Boulton AJ, Armstrong DG, Albert SF, Frykberg RG, Hellman R, Kirkman MS (2008). Comprehensive foot examination and risk assessment: a report of the task force of the Foot Care Interest Group of the American Diabetes Association, with endorsement by the American Association of Clinical Endocrinologists. Diabetes Care.

[21] Parisi MCR, Moura Neto A, Menezes FH, Gomes MB, Teixeira RM, Oliveira JEP (2016). Baseline characteristics and risk factors for ulcer, amputation and severe neuropathy in diabetic foot at risk: the BRAZUPA study. Diabetol Metab Syndr.

[22] Tesfaye S, Selvarajah D (2012). Advances in the epidemiology, pathogenesis and management of diabetic peripheral neuropathy. Diabetes Metab Res Rev..

[23] Boulton AJM, Vinik AI, Arezzo JC, Bril V, Feldman EL, Freman R, American Diabetes Association (2005). Diabetic neuropathies: a position statement by the American Diabetes Association. Diabetes Care.

[24] Malik RA, Veves A, Tesfaye S, Smith G, Cameron N, Zochodne D, Lauria D (2011). Small fibre neuropathy: role in the diagnosis of diabetic sensorimotor polyneuropathy. Diabetes Metab Res Rev.

[25] Coffey L, Mahon C, Gallagher P (2016). Perceptions and experiences of diabetic foot ulceration and foot care in people with diabetes: a qualitative meta-synthesis. Int Wound J..

[26] Apelqvist J, Bakker K, Van Houtum WH, Nabuurs-Franssen MH, Schaper NC (2000). International consensus and practical guidelines on the management and the prevention of the diabetic foot: international Working Group on the Diabetic Foot. Diabetes Metab Res Rev..

